# ICTV Virus Taxonomy Profile: Rhabdoviridae 2022

**DOI:** 10.1099/jgv.0.001689

**Published:** 2022-06-20

**Authors:** Peter J. Walker, Juliana Freitas-Astúa, Nicolas Bejerman, Kim R. Blasdell, Rachel Breyta, Ralf G. Dietzgen, Anthony R. Fooks, Hideki Kondo, Gael Kurath, Ivan V. Kuzmin, Pedro Luis Ramos-González, Mang Shi, David M. Stone, Robert B. Tesh, Noël Tordo, Nikos Vasilakis, Anna E. Whitfield, ICTV Report Consortium

**Affiliations:** 1 School of Chemistry and Molecular Biosciences, University of Queensland, St Lucia, QLD 4072, Australia; 2 Brazilian Agricultural Research Corporation, Cruz das Almas-BA, 44380-000, Brazil; 3 Consejo Nacional de Investigaciones, Científicas y Técnicas (CONICET) and Instituto Nacional de Tecnología Agropecuaria (INTA), Argentina; 4 CSIRO Health and Biosecurity, Geelong, VIC 3220, Australia; 5 University of Washington, Seattle, WA 98105, USA; 6 Queensland Alliance for Agriculture and Food Innovation, University of Queensland, St Lucia, QLD 4072, Australia; 7 Animal and Plant Health Agency Addlestone, Surrey KT15 3NB, UK; 8 Institute of Plant Science and Resources, Okayama University, Kurashiki, 710-0046, Japan; 9 Western Fisheries Research Center, Seattle, WA 98115, USA; 10 University of Texas Medical Branch, Galveston, TX 77555, USA; 11 Instituto Biológico, São Paulo, Brazil; 12 Sun Yat Sen University, Guangzhou, Guangdong, PR China; 13 Centre for Environment, Fisheries and Aquaculture Science, Weymouth, DT4 8UB, UK; 14 Institut Pasteur de Guinée, Gamal Abdel Nasser University, Conakry, Guinea; 15 Department of Entomology and Plant Pathology, North Carolina State University, Raleigh NC 27606, USA

**Keywords:** ICTV Profile, taxonomy, *Rhadboviridae*

## Abstract

The family *Rhabdoviridae* comprises viruses with negative-sense (−) RNA genomes of 10–16 kb. Virions are typically enveloped with bullet-shaped or bacilliform morphology but can also be non-enveloped filaments. Rhabdoviruses infect plants or animals, including mammals, birds, reptiles, amphibians or fish, as well as arthropods, which serve as single hosts or act as biological vectors for transmission to animals or plants. Rhabdoviruses include important pathogens of humans, livestock, fish or agricultural crops. This is a summary of the International Committee on Taxonomy of Viruses (ICTV) Report on the family *Rhabdoviridae*, which is available at ictv.global/report/rhabdoviridae.

## Virion

**Fig. 1.**
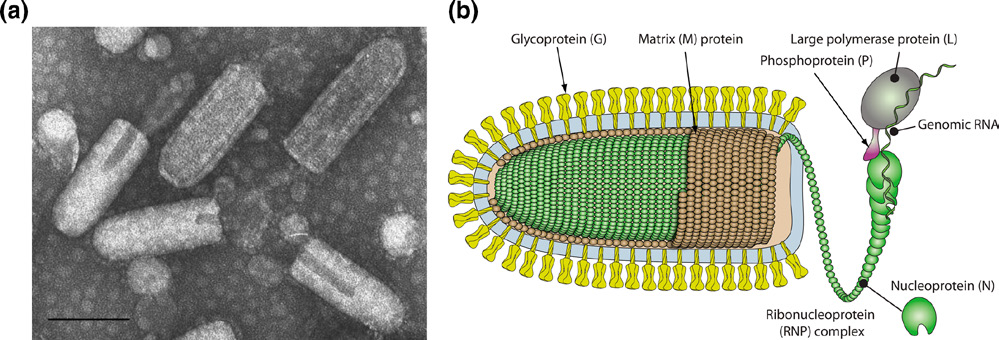
(a) Negative-contrast electron micrograph of vesicular stomatitis Indiana virus particles. The bar represents 100 nm (courtesy of P. Perrin). (b) Schematic illustration of a rhabdovirus virion and ribonucleocapsid structure. Unravelling of the RNP is solely illustrative to show its association with L and P more clearly (courtesy of P. Le Mercier).

Virions are usually enveloped and bullet-shaped or bacilliform (i.e. with two rounded ends) and contain five structural proteins (Table 1, Fig. 1). The nucleocapsid protein (N), the large multi-functional RNA-directed RNA polymerase (L) and the polymerase-associated phosphoprotein (P), together with the RNA genome, form the ribonucleoprotein (RNP) complex. The nucleocapsid is encased in the matrix protein (M) layer, which also interacts with the envelope containing the transmembrane glycoprotein (G). Rhabdoviruses assigned to the genera *Dichorhavirus* and *Varicosavirus* lack an envelope.

**Table 1.** Characteristics of members of the family *Rhabdoviridae*

Example:	vesicular stomatitis Indiana virus (AF473864), species *Vesiculovirus indiana*, genus *Vesiculovirus*
**Virion**	Bullet-shaped or bacilliform particle 100–430 nm in length and 45–100 nm in diameter comprising a helical nucleocapsid surrounded by a matrix layer and a lipid envelope. Some rhabdoviruses have non-enveloped filamentous or bacilliform virions
**Genome**	Negative-sense RNA of 10–16 kb (unsegmented or bi-segmented)
**Replication**	Ribonucleoprotein (RNP) complexes containing full anti-genomic RNA are generated and serve as templates for synthesis of nascent RNP complexes containing genomic RNA
**Translation**	Capped and polyadenylated mRNAs transcribed processively from each gene (3′ to 5′), sometimes containing multiple ORFs
**Host range**	Vertebrates, invertebrates and plants; many vertebrate and plant rhabdoviruses are arthropod-borne
**Taxonomy**	Realm *Riboviria*, kingdom *Orthornavirae*, phylum *Negarnaviricota*, subphylum *Haploviricotina*, class *Monjiviricetes*, order *Mononegavirales;* the family includes 3 subfamilies >44 genera and >274 species

## Genome

**Fig. 2.**
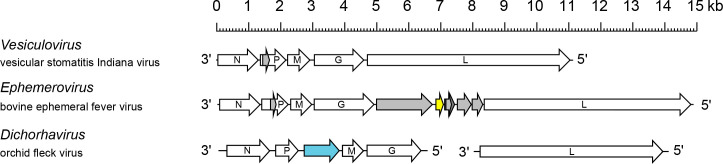
Schematic representation of several rhabdovirus genome organizations, exemplifying variations in architecture and the number and location of accessory genes. Arrows indicate the position of long ORFs. Other alternative ORFs occur in some genes; only ORFs (≥180 nt) that appear likely to be expressed are shown. ORFs encoding viroporin (yellow) and movement proteins (blue) are shown.

Rhabdovirus negative sense (−) RNA genomes are 10–16 kb (Fig. 2) [1]. Almost all rhabdovirus genomes are unsegmented, but rhabdoviruses with bi-segmented genomes are also known [2]. Terminal non-coding regions are partially complementary. Genomes usually encode five major structural proteins but may also encode additional (accessory) proteins, either in additional genes or as alternative ORFs within the structural protein genes [1,3].

## Replication

Rhabdovirus replication generally occurs in the cytoplasm following receptor-mediated endocytosis. Primary transcription is initiated from the incoming (−)RNP complex by the RNA-directed RNA polymerase (RdRP). Stop–start transcription occurs 3′ to 5′ using gene start and gene end sequences to generate polyadenylated mRNAs. Replication is initiated by the RdRP from a single promoter at the 3′-end, ignoring gene start and gene end sequences to generate a (+)RNP. This is the template for nascent (−)RNPs, which are assembled with M and G into enveloped virions. Budding can occur at either the plasma membrane or internal membranes. Some plant rhabdoviruses replicate in the nucleus.

## Taxonomy

Current taxonomy: ictv.global/taxonomy. Viruses assigned to each genus form a monophyletic clade based on phylogenetic analyses of L protein sequences and usually have similar genome organizations, including the number and locations of accessory genes. Rhabdoviruses have been isolated from a wide range of vertebrates and plants; many have been isolated from arthropods [2,4, 5]. The subfamily *Alpharhabdovirinae* includes >30 genera for viruses infecting only vertebrates, only invertebrates, or vertebrate hosts and arthropod vectors. These viruses have been referred to informally as dimarhabdoviruses (dipteran and mammalian rhabdoviruses), but various members may infect birds, reptiles, amphibians, non-dipteran insects, ticks, or nematodes. The subfamily *Betarhabdovirinae* includes >five genera for viruses infecting plant hosts and arthropod vectors. These include viruses with bi-segmented genomes (genera *Dichorhavirus* and *Varicosavirus*) and rod-shaped, non-enveloped virions (genus *Varicosavirus*). The subfamily *Gammarhabdovirinae* includes the genus *Novirhabdovirus* for viruses infecting teleost fish. Several other genera are not assigned to a subfamily; viruses assigned to these genera have been detected by high-throughput sequencing of invertebrate metagenomes.

## Resources

Full ICTV Report on the family *Rhabdoviridae*: ictv.global/report/rhabdoviridae.

## Abbreviations

G: glycoprotein
L: large polymerase protein
M: matrix protein
N: nucleocapsid protein
P: phosphoprotein
RdRP: RNA-directed RNA polymerase
RNP: ribonucleoprotein
